# Composition of uroliths in a tertiary hospital in South East Nigeria

**DOI:** 10.4314/ahs.v18i2.29

**Published:** 2018-06

**Authors:** Ijeoma A Meka, Martin C Ugonabo, Samuel O Ebede, Ezra O Agbo

**Affiliations:** Department of Chemical Pathology, University of Nigeria Teaching Hospital, Ituku-Ozalla, PMB 01129, Enugu, Enugu State, Nigeria

**Keywords:** Uroliths, calcium oxalate, chemical composition, struvite, stone, calculi

## Abstract

**Background:**

Urolithiasis affects primarily the urinary tract and complications as debilitating as renal failure may develop. Determining the chemical composition of uroliths can aid management and prevention of recurrence in patients.

**Objective:**

To determine the chemical composition and anatomical distribution of uroliths in Nigeria.

**Methods:**

This descriptive cross-sectional study was conducted between March 2014 and February 2016, in a tertiary hospital in Nigeria. We reviewed the outcomes of uroliths of adult patients sent to our laboratory for chemical analyses. Samples were analyzed using simple qualitative tests.

**Results:**

52 adult patients were included with a mean age (SD) of 46.6 (12.6) years. Males (76.9%) were more affected than females (23.1%). For both sexes, highest occurrence of stones was in bladder (85.7%). Calcium-containing stones had the highest occurrence (85.2%) and predominated in the renal, ureter and urethra, followed by struvite stones (59.5%). In the bladder, struvite stones were predominant (85.8%), with calcium-containing stones accounting for 71.4%.

**Conclusion:**

This study showed that struvite and calcium phosphate-containing stones constitute majority of uroliths in our setting with low occurrence of calcium oxalate stones. This indicates that urinary tract infection most likely plays a substantial role in the formation of uroliths in Nigerians. Modern methods of stone analysis is advocated to further define management options.

## Introduction

Urolith simply means a calculus or stone in the urinary tract and hence comprises renal, ureteral, bladder and urethral calculi. These are solid masses formed in the urinary tract from minerals in urine. They represent an age long medical condition which places an appreciable disease burden on the global populace. The prevalence and incidence of stone diseases particularly kidney stones, is reported to be increasing worldwide and these increases are seen across sex, age and race.^1^ In the United States in 2012,^2^ kidney stones were reported to affect 1 in 11 people representing a marked increase in stone disease particularly in Black, non-Hispanic and Hispanic individuals. In Germany,^3^ an increase in prevalence of urolithiasis was reported from 4% in 1979 to 4.7% in 2001 with the recurrence rate estimated to be 42%. In a tertiary health care centre in Nigeria, between 1980 and 1986,^4^ an increase in prevalence of urolithiasis was reported. Two independent studies also reported incidences of 13^5^ and 6.3^6^ per 100,000 patients in two different tertiary health care centres in Nigeria.

With these documented increases in prevalence of stone disease, determination of composition of uroliths is of great importance to provide insight to the etiology of the stone and hence the management and prevention of recurrence. Stone composition varies greatly but calcium oxalate, calcium phosphate, uric acid, struvite (magnesium ammonium phosphate), and cystine are the most common urinary stone types. Some of the risk factors associated with urolithiasis include^7^,^23^,^27^,^29^ dehydration, family history, obesity, prior occurrence, urinary tract infection, metabolic disorders like gout, hyperparathyroidism, drugs like diuretics, among others.

It has been documented that stone disease is commoner in Whites than Blacks^8^,^9^ but the associated pain and morbidity may not differ. Hence there is need to regularly update the frequency, anatomical distribution, and composition of stones in persons at-risk of forming urinary stones in the Black population especially in resource-limited settings like Nigeria where treatment is still predominantly by open surgery. This is essential to limit recurrence rates and frequency of surgical interventions with the attendant pain and possible complications.

In South-East, Nigeria, the last and only available attempt at this characterization was 32 years ago by Mbonu et al^5^, hence the necessity of this study to determine the anatomical distribution and composition of uroliths in a sub-population of Black patients.

## Materials and methods

### Study location

This was a 3½-year descriptive cross-sectional study conducted at the department of Chemical Pathology, University of Nigeria Teaching Hospital (UNTH), Enugu, a tertiary health institution in South-East Nigeria, between March, 2014 and February, 2016. UNTH is a 576 bedded hospital serving Enugu, Anambra and Abia states, all states in the South-East geopolitical zone of Nigeria. The inhabitants of these states are mainly indigenes of Igbo extraction, whose main occupation are farming and trading with a small proportion subsisting as civil servants.

### Study population

The study was carried out using uroliths from consecutively selected consenting adult patients aged 18 years and above, who had surgery for urinary tract stone removal and the stone(s) sent for chemical analysis within the study period.

### Study design

Prior to chemical analysis, the stones were washed with distilled water, air dried and weighed.

Each stone was then cut into two and examined for a nucleus. Presence of a nucleus required separate analysis of nucleus and periphery. The stone was then crushed using a porcelain mortar and pestle and the powdered form subjected to chemical analysis using the McIntosh and Salter method^10^. Patients' records were carefully reviewed for age, sex, race and location of stones.

### Inclusion criteria

All consenting adult patients aged 18 years and above who had surgery for urinary tract stone and the stones sent for chemical analysis during the study period.

### Exclusion criteria

Paediatric patients, adult patients with gall-bladder stones and patients declining consent.

### Ethical considerations

Written informed consent was obtained from participants and ethical clearance obtained from UNTH Health Research and Ethics Committee with reference number UNTH/CSA/329/5.

### Statistical analysis

Data was analyzed using SPSS version 20 and reported as frequencies for categorical variables, and mean (standard deviation) for continuous variables. Chi-square test was used to compare categorical variables. A p-value <0.05 was used to denote statistical significance.

## Results

During the study period, 54 uroliths were received from 52 patients aged 18 years and above (one patient had 3 stones from different anatomical locations; renal, ureter and bladder). All participants were Blacks from South East, Nigeria. Mean age (SD) in years was 46.6 (12.6).

More males (76.9%) were affected than females (23.1%) with a Male to Female ratio of 3.3:1.

**Fig 1:**
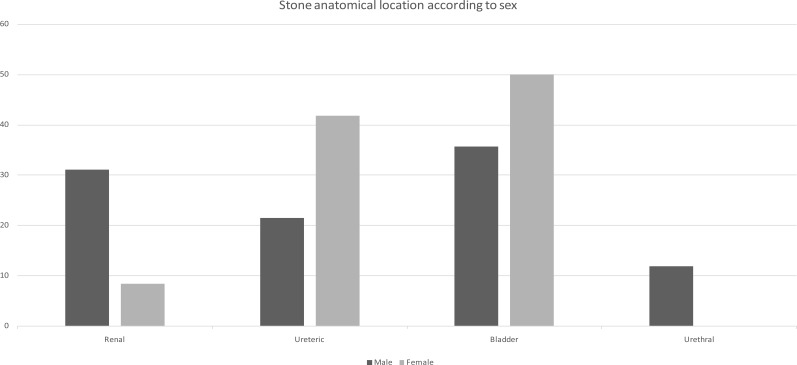
Stone anatomical location according to sex

Patients who fell within the 30 – 49 years age group had the highest number of stones in both males and females (50% each). The age and sex distribution of the urinary stones is as shown in Table 1. The anatomical location of the urinary stone varied according to sex and age groups of the patients though these variations were not statistically significant, χ2=3.69(3, N=52) p=0.3 and χ2= 8.64 (3, N=52) p=0.47 respectively. In females, 50% of the stones occurred in the bladder and 41.7% were ureteric stones (Fig 1). In males, 35.7% were bladder stones followed by renal stones (31.0%), and ureteric stones (21.4%). All the age groups, except those within the 50 – 69 age group, had the highest number of stones occurring in the bladder. The anatomical locations of the stones according to sex is as shown in Fig 1 while Table 2 shows the anatomical locations according to age groups.

**Table 1:** Age and Sex distribution

Age group (years)	Male Number (%)	Female Number (%)	Total Number (%)
18 – 29	3 (7.5)	2 (16.7)	5 (9.6)
30 – 49	20 (50.0)	6 (50.0)	26 (50.0)
50 – 69	14 (35.0)	4 (33.3)	18 (34.6)
70 and above	3 (7.5)	0 (0.0)	3 (5.8)
Total	40 (100)	12 (100)	52 (100)

**Table 2:** Stone anatomical location according to age groups

Age group(years)	Anatomical location			
	Renal	Ureter	Bladder	Urethra
	Number (%)	Number (%)	Number (%)	Number (%)
18 –29	−	−	3 (5.8)	2 (3.8)
30 – 49	5 (9.6)	8 (15.4)	12 (23.1)	3 (5.8)
50 –69	9 (17.3)	6 (11.5)	3 (5.8)	−
70 and above	−	−	3 (5.8)	−

NB: Total above 100% due to individuals with multiple stones

All stones, 52 (100%) were found to be of mixed composition, no pure stone was encountered. Calcium-containing stones had the highest occurrence (85.2%) as all the stones analyzed with the exception of magnesium ammonium phosphate (Struvite) stones occurring alone contained calcium.

Interestingly, among the calcium-containing stones, calcium phosphate stones had the highest occurrence of 63.1% either alone or in combination. Stones containing Magnesium Ammonium Phosphate either alone or in combination constituted 59.5%. No uric acid, xanthine or cystine stones were found. Composition of bladder stones having the highest occurrence in both sexes was analyzed separately. Magnesium ammonium phosphate either alone or in combination accounted for the highest occurrence (85.8%). Calcium-containing stones accounted for 71.4% among which 52.5% contained calcium phosphate in combination with other ions. Table 3 shows the chemical composition of all the stones while Table 4 shows the chemical composition of the bladder stones.

**Table 3:** Chemical composition of Stones

S/N	Constituents	Number (%)
1	Calcium oxalate + Magnesium Ammonium Phosphate	6 (11.5)
2	Calcium phosphate + Magnesium Ammonium Phosphate	24 (46.2)
3	Calcium oxalate + Calcium phosphate + Magnesium	1 (1.9)
4	Calcium phosphate + Ammonia	5 (9.6)
5	Magnesium Ammonium Phosphate alone	8 (15.4)
6	Calcium + Ammonia + Magnesium	5 (9.6)
7	Calcium phosphate alone	2 (3.8)
8	Calcium phosphate + Carbonate + Ammonia	1 (1.9)
9	Calcium phosphate + Magnesium	1 (1.9)
10	Calcium + Ammonia	1 (1.9)

NB: Total above 100% due to individuals with multiple stones

**Table 4:** Chemical composition of bladder stones

S/N	Constituents	Number (%)
1	Calcium oxalate + Magnesium Ammonium Phosphate	3 (14.3)
2	Calcium phosphate + Magnesium Ammonium Phosphate	9 (42.9)
3	Calcium oxalate + Calcium phosphate + Magnesium	1 (4.8)
4	Calcium phosphate + Ammonia	1 (4.8)
5	Magnesium Ammonium Phosphate alone	6 (28.6)
6	Calcium + Ammonia + Magnesium	1 (4.8)
	Total	21 (100)

## Discussion

The number of uroliths found in this study within the study period corroborates previous findings that urinary tract stones are commoner in Whites than in Blacks^8^,^9^. This is further corroborated by a study done in Ghana^11^ which reported a total of fifty-one patients with newly diagnosed upper urinary tract stones seen over an eight (8) year period, giving an incidence of 2 per 100,000. Again, it is generally accepted that urinary calculi occur more in males and the greater percentage of affected males seen in this study corroborates this. The male to female ratio of 3.25:1 seen in this study is similar to 4:1 recorded by Monu^6^ in Benin, Nigeria. Studies in Northern Nigeria^12^ and Kenya^13^ also recorded preponderance of the disease in males. The age group with the highest percentage of stone formers in this study, 30 – 49 years is similar to the 31– 40 year age group recorded by Mbonu et al^5^ and 30 –40 year age group recorded in Nairobi^14^. And also similar to the peak age of 38 years recorded by Rahman *e*t al^15^, 30 – 39 age group recorded in Saudi Arabia^16^ and 30 – 44 year age group recorded by Channa et al^17^. The reason for the high incidence in this age group is not clear, but urinary tract infections, dietary factor and obesity may be strong possibilities. Individuals in this age group are more likely to be sexually active and there is evidence^18^,^19^ to suggest that sexual activity predisposes to urinary tract infection in both pre- and post-menopausal women. High intake of calorie-rich drinks, low water intake, excess calorie intake consequently results in obesity which is also associated with stone formation^20^,^21^. Recent studies have indicated an increase in the prevalence of obesity and overweight in Nigeria^22^,^23^ and Desalu et al^24^ further recorded the highest prevalence to be in the 40 – 49 years age group.

Bladder calculi were of the highest frequency in both sexes. This corroborates the findings of Mbonu et al^5^ previously done in the same region of Nigeria, and Jarrar et al^16^, which also recorded highest frequency of stones in the bladder and further buttresses the fact that bladder stones are still common in developing countries unlike in the western world^25^. This however differs from the studies in Maiduguri, Northern Nigeria,^12^ and Kenya^13^ which recorded higher frequency of stones in the upper urinary tract.

The composition of the bladder stones in this study shows predominance of magnesium ammonium phosphate (struvite) stones, also known as infection stones. These infection stones are usually secondary to urinary tract infection with a urease-producing organism, such as *Proteus spp* or *Klebsiella spp*. Urease catalyzes the hydrolysis of urea to produce ammonia, elevating the urine pH. As mentioned above, urinary tract infections may be secondary to sexual intercourse. Moreover, benign prostatic enlargement, urethral strictures, neurogenic bladder dysfunction and pelvic organ prolapse contribute to bladder outlet obstruction, causing urinary stasis as well as increased infection rates.^26^ In women, the short length of the urethra also makes urinary tract infections common.

This finding corroborates earlier findings^5^,^6^ which recorded that a significant number of stone diseases in Nigeria were secondary to obstruction, infection and immobilization. Patients with infection stones have a high incidence of new stone growth and persistent infection, especially if residual stone fragments remain, hence the importance of complete eradication of these organisms needs constant emphasis.^27^

The high occurrence of calcium-containing stones is also similar to that recorded in other studies.^12^,^16^,^28^

However, the low occurrence of calcium oxalate stones in this study differs from the usual picture seen in Caucasians^29^,^30^ where calcium oxalate stones form the greater percentage of stones. This is not surprising due to the correspondingly high prevalence of infection stones among the study population. As mentioned earlier, urease activity produces ammonia and renders the urine alkaline, which in turn favours the formation of calcium phosphate stones.

The risk factors for calcium stone formation^31^,^32^,^33^,^34^ range from hypercalciuria, chronic low urine output, hyperparathyroidism, to renal tubular acidosis. There is evidence that the degree of hypercalciuria is worsened by high dietary sodium intake, high animal protein intake, and loop diuretics. A study of 120 Italian hypercalciuric calcium oxalate stone patients has demonstrated that a diet with normal calcium, low sodium, and low animal protein resulted in reduced incidence of calcium stones compared with those on a low-calcium diet.^35^

With abundance of health tips freely available on social media currently, many stone formers may feel the need to decrease calcium in their diet due to the predominance of calcium in urinary tract stones thereby worsening the process. Hence, proper individualized patient counselling and health education based on the stone composition of an individual is advocated in this regard.

Apart from chemical analysis of stones, management should also include urine culture and anti-microbial sensitivity testing which should be obtained every 1 or 2 months during the first year, and at regular intervals thereafter. Urine electrolytes and other tests indicated by the patient's general condition may also be necessary.

## Conclusion

Calcium and struvite stones constitute the commonest components of uroliths in this study. Hence, effective clinical management with complete eradication of the causative organisms, proper individualized patient counselling with emphasis on diet and lifestyle measures are necessary for prevention of stone recurrence and generally improving the quality of life of the patients. This study is based on simple qualitative chemical analysis which is cost effective for patients, feasible and adaptable to the level of manpower available in a developing country.

However, with the existence of more modern methods like Fourier-transform infrared spectroscopy (FT-IR) and X-ray diffraction (XRD) which are beyond the capacities existent in resource-limited countries like Nigeria, more work is needed in this area in order to further highlight steps which may be necessary in effective management.
